# Are A1C levels at the moment of DM diagnosis associated with renal outcomes?

**DOI:** 10.1186/1758-5996-7-S1-A33

**Published:** 2015-11-11

**Authors:** Priscila Aparecida Correa Freitas, Ana Laura Pimentel, Gabriela Cavagnolli, Joíza Lins Camargo

**Affiliations:** 1 IPA, Porto Alegre, Brazil

## Background

Hyperglycemia is a risk factor to renal disease (RD). It is recommended that patients with diabetes mellitus (DM) are screened to RD at the moment of the diagnosis, by urinary albumin levels. Glycated hemoglobin (A1C) is one of the available diagnosis test to DM and also a known predictor factor to RD.

## Objectives

To determine if A1C levels at the moment of DM diagnosis are associated with future renal outcomes measured by urinary albumin excretion rate.

## Materials and methods

This prospective study evaluated 269 patients screened to DM type 2 at a university hospital between 2008 and 2009. All patients performed an oral glucose tolerance test (OGTT), fasting glucose (FG), urinary albumin and A1C measured by colorimetry, immunoturbidimetry (Advia 1800, Siemens Diagnostica) and HPLC (2.2 Tosoh Plus A1C, Tosoh Corporation), respectively. They were identified with DM according to ADA criteria. Between 2010 and 2012, the patients returned and were re-evaluated. Renal outcomes were measured by urinary albumin levels in the follow up, according with KDIGO guidelines. Poisson regression with robust standard errors was performed in those with DM diagnosis, considering the worsening of renal function, measured by urinary albumin levels, as dependent variable and A1C, FG, 2h plasma glucose after OGTT (G2h) levels, age and hypertension as independent variables. Statistical analysis was performed by SPSS 20.0 and p <0.05 was considered as statistically significant.

## Results

Of the 269 patients analyzed, 71 (26.4%) had DM diagnosis (44 women, age of 57±12 yrs.). After a follow up of 30.2±7.0 months, there was no association between A1C, FG nor G2h basal levels with renal outcomes, adjusted for age and hypertension (p >0.05). Only age was risk factor (p <0.001; relative risk 1.074 [1.035-1.114] to the worsening of renal function, where the increase of one year in age was associated with 7.4% increase in the risk to renal outcomes.

## Conclusion

In this small cohort, A1C levels at the diagnosis of DM showed no association with future urinary albumin levels. However, the short follow up time and small number of patients may have been a limitation of this study.

